# Clinical comparison of simultaneous integrated and sequential boost IMRT of tumor bed after breast conserving surgery

**DOI:** 10.4314/ahs.v25i2.20

**Published:** 2025-06

**Authors:** Minghai Shao, Caiping Jiang, Yunxiang Yao, Weifang Yang, Haijian Jia, Yichao Shen, Yanli Wang, Bin Yu

**Affiliations:** Key Laboratory of Minimally Invasive Techniques & Rapid Rehabilitation of Digestive System Tumor of Zhejiang Province, Department of Radiation Oncology, Taizhou Hospital of Zhejiang Province affiliated to Wenzhou Medical University, Linhai 317000, Zhejiang Province, China

**Keywords:** Clinical outcomes, breast cancer, SIB-IMRT, SEQ-IMRT

## Abstract

**Background:**

To explore the clinical effect compare between the simultaneous-integrated boost and sequential-boost intensity modulated radiotherapy in early breast cancer patients after breast conserving surgery.

**Methodology:**

According to the different postoperative radiotherapy methods, 66 early breast cancer patients after breast-conserving surgery were divided into two groups, 34 in the simultaneous-integrated boost group and 32 in the sequential-boost intensity group. Adverse reactions, cosmetic effects, local recurrence rate, progression-free survival and overall survival rates were compared.

**Results:**

No statistical significance in the incidence of acute skin reaction, advanced skin reaction, myelosuppression, radiation pneumonia and other adverse reactions between two groups were observed. The fine rate of simultaneous-integrated boost group was slightly better than the sequential-boost intensity group. And the follow-up rate of the two groups was 100%. The progression-free survival rate of the simultaneous-integrated boost group and the sequential-boost intensity group were 100% and 96.88%, respectively; The overall survival rates of the two groups were all 100%; One patient in the sequential-boost intensity group had recurrence (3.13%), while none in the simultaneous-integrated boost group. No significant difference in local recurrence rate, progression-free survival and overall survival rates in the two groups were found.

**Conclusion:**

Simultaneous-integrated boost ensures breast appearance and survival without added radiotherapy side effects vs sequential-boost intensity.

## Introduction

Breast cancer is the commonest cancer in women around the world 1. It is the second principal reason for cancer death among global women, accompanied by a slight but stable increase in its incidence over the last decade 2,3. In China, 17% of all female malignant tumors were breast cancer, and it is becoming younger^4^. Therefore, to prevent and control breast cancer as well as improve the patients' survival conditions have been urgent. Early detection and early treatment is the key to breast cancer treatment, which is of importnce to increase the survival rate while reducing the mortality rate. With the improvement of diagnosis level, people begin to pay attention to the aesthetic effect and life quality after breast cancer operation, the breast-conserving surgery (BCS) has been widely concerned. BCS includes breast-conserving surgery and adjuvant radiotherapy, which becomes one kind of the standard protocol for local breast cancer treatment. BCS can retain the normal breast tissue, and has the advantages of less invasion and more aesthetic 5. The commonly used radiotherapy regimen after BCS is whole breast irradiation therapy (WBRT). BCS plus the adjuvant WBRT showed the equivalent local traditional control and overall survival rates compared with mastectomy^6^. To further promoted the local control of young patients or high-risk tumor patients, the additional boost irradiation was used to control the recurrence rate of tumor bed 7. Whereas, before a long-term study found that the use of uneven radiation and photon beam boosts in tumor-bed boosts bring patients poor cosmetic effect^8^. Intensity-modulated radiation therapy (IMRT) can improve the consistency and uniformity of the dose, reducing high dose for hearts and lungs at the cost of enhancing total low doses, and it was proved to improve acute skin toxicity, hence has been used adjunctively for WBRT 9. According to the tumor bed boost timing, IMRT can be classified into the sequential boost (SEQ)-IMRT and the simultaneous integrated boost (SIB)-IMRT, with the former applied after the WBRT, the latter is applied simultaneously with WBRT^10^. A previous meta-analysis reported that in view of reduction of the acute side effects, IMRT is a viable treatment option for women undergoing external beam RT after BCS 11.

This study compared the efficacy, adverse reactions and cosmetic effects of synchronous IMRT and sequential IMRT after BCS, in order to provide reference for the selection of radiotherapy after BCS for breast cancer patients.

## Material and Methods

### Study population

Retrospective analysis was performed on the clinical data of 66 patients diagnosed with early breast cancer and underwent BCS during December 2014 to June 2016.

Inclusion criteria: (1) Clinically and pathologically confirmed early breast cancer grade I or grade II 12; (2) Age ≥ 18 years old; (3) Breast-conserving surgery was performed in our hospital; (4) No radiotherapy contraindications; (5) Karnofsky performance status (KPS) score ≥ 80; (6) Postoperative resection margins were negative, and the maximum diameter of the primary tumor ≤ 3 cm; (7) Mammogram examination after breast conserving indicated no extensive sandy calcification of the breast; (8) Written informed consent. Exclusion criteria: (1) had received radiotherapy or other treatment within 3 months prior to surgery; (2) Failure to follow the radiotherapy regimen or failure to follow up; (3) Benign and malignant tumors in other sites; (4) Pregnant or lactating women; (5) Incomplete function of other organs; (6) Combined with immune system or coagulation system disorders; (7) Cognitive dysfunction or mental illness; (8) Presence of distant metastasis; (9) Incomplete clinical data.

A total of 66 patients diagnosed with early breast cancer were included in this study according to the above enrolled standards. According to the different postoperative radiotherapy protocol, these patients were classified into the SIB-IMRT group (n=34) and the SEQ-IMRT group (n=32). The age range of 66 selected patients was 18 to 65 years.

### Treatment planning

All enrolled patients agreed to receive breast-conserving surgery and postoperative treatment, and underwent axillary lymph node dissection. 2-6 titanium clamps were indwelled in the intraoperative cavity, and the lower margins were negative, without vascular and lymphatic vessel enveloping infiltration. IMRT was performed 4-8 weeks after surgery.

#### (1) Computed tomography (CT) for location simulation

The patient was asked to take a supine position, on a breast bridge (breast positioning board) with both arms abducted and elevated above the head. Axial CT scanning range was 5 cm below the cricothyroid membrane to the lower edge of the lung, and the thickness of the reconstructed layer was 5 mm.

#### (2) Delineation of target volume and organs at risk (OARs)

Based on the guidelines of ESTRO 13 to delineated the clinical target volume (CTV) for whole breast. The expansion of CTV for 0.5 cm around the tumor bed in all directions except the skin formed the planning target volume (PTV-breast), which its anterior margin was 0.3 cm from the skin. Gross tumor volume (GTV) should be delineated on CT images, and the contour was outlined according to the external placement of the titanium clip, which was named as the target volume of the boost tumor bed (GTV-t). And CTV-boost was expanded 1-1.5 cm on the basis of the GTV-t, which should not exceed the scope of the whole breast CTV. Considering the influence of positioning error and respiratory activity, the planned target volume of the tumor bed was expanded by 0.5 cm on the basis of CTV-boost to form the PTV-boost, which did not exceed the PTV range of the whole breast.

Depicted OARs include the ipsilateral and contralateral breast, the heart and spinal cord, as well as the ipsilateral and contralateral lungs. The heart does not include pericardial adipose tissue, but is delineated from the trunk of pulmonary to near the diaphragm. Two experienced radiologists reviewed and modified the delineation of the target volume and OARs.

### Irradiation therapy plans

#### SIB-IMRT group

Using 6MV X-ray, 6-8 irradiation fields were determined based on the target area, size and shape of the affected side mammary gland. Prescription dose setting: (1) PTV-breast: 50.4 Gy delivered in 28 fractions; (2) PTV-boost: 60 Gy delivered in 28 fractions. The two parts are carried out at the same time.

#### SEQ-IMRT group

The whole breast irradiation is the same as SIB-IMRT group, which adopts 6 MV X-ray and designs 6-8 irradiation fields according to the shape, size and target area of the affected side breast. Prescription dose setting: (1) PTV-breast: 50 Gy delivered in 25 fractions; (2) PTV-boost: 10 Gy delivered in 5 fractions. The tumor-bed boost followed the whole breast irradiation.

The two groups received 50 Gy / 25 F in ipsilateral upper and lower clavicular region with or without radiotherapy. Reverse intensity modulated radiation therapy (RRT) was applied to the Varian trilogy linear accelerator in the United States. The planning system was Eclipse10.0. The goal of both plans is to use 95% of the prescribed dose to cover 95% of the target-volumes (PTV-breast and PTV-boost). Though the OARs dose constraints were prescribed, a low dose was needed possible. There were dose limit specified for OARs: (1) the mean dose of the contralateral breast and lung should be less than 5 Gy, (2) < 10% heart volume allowed exceeds 30 Gy, and (3) < 20% ipsilateral lung allowed exceeds 20 Gy.

### Evaluation index of treatment effect

#### (1) Postoperative adverse reactions

Acute radiation injury classification standards of RTOG were adopted 14: skin, hematology and pulmonary toxicity were mainly observed. Acute skin grading criteria: Grade 0 ∼ IV. The symptoms are as follows: Grade 0 was no change; Grade I was follicular dark erythema; Grade II was dry peeling, blisters, itching; Grade III was wet peeling, ulcer; Grade IV was exfoliating dermatitis, necrosis. Acute radioactive hematological toxicity classification criteria: leukocyte (1 000/m3): ≥ 4.0 was grade 0; 3.0 ∼ < 4.0 was Grade I; 2.0 ∼ < 3.0 was grade II; 1.0 ∼ < 2.0 was grade III; < 1.0 was grade IV. Chest CT examination combined with symptoms and signs was used to evaluate the pulmonary toxicity of the patients. The grading criteria of acute radiation pneumonia were as follows: no change, mild dry cough, dyspnea after activity, dyspnea at rest, continuous oxygen inhalation and complete bed rest.

#### (2) Cosmetic effect of breast

The cosmetic effects of breast between the two groups was compared 1 month after radiotherapy. Qualitative cosmetic results evaluation contains the shape, size, fibrosis of the operated breast and appearance of the surgical scar, the skin color, shape and location of the nipple and areola. These evaluations were rated as excellent, good, fair, and poor according to the Harvard scale 15,16. The symptoms of each grade are as follows: Excellent: No visible treatment sequelae, the appearance of both breasts is the same. Good: There is mild pigmentation, local telangiectasia, surgical scar visible. General: mammary gland appearance has obvious deformation, nipple shift, there is obvious telangiectasia. Poor: the breast has serious retraction or serious fibrosis and other serious changes in appearance. Fine rate = (excellent + good) cases/total cases *100% 15,17.

#### (3) Local control rate, progression-free survival and overall survival rates

The first follow-up was 2 months after radiotherapy, and once every 3 months within the 2 years after radiotherapy. In the 3-5 years, the patients were followed up every 6 months. Observed the 5-year overall survival rate and progression-free survival rate of the two groups and compared the difference.

### Statistical analysis

Statistical Product and Service Solutions (SPSS) 23.0 software (IBM, Armonk, NY, USA) was performed for the data analysis. Statistical data were represented as the number of cases and the rate (%), while the measurement data were expressed by mean ± standard deviation (x̅±s). t-test and x^2^ test was conducted for the comparison between groups. P < 0.05 was used to assess statistical significance.

## Results

### Baseline characteristics

In the current study, we enrolled 66 patients who treated with IMRT after BCS during December 2014 to June 2016. Patients were diagnosed with stage I or II breast cancer based on the 2009 7th edition of the AJCC 18. All patients received lumpectomy with axillary lymph node dissection (ALND) or sentinel lymph node dissection (SLND) and ensured tumor-negative margins in a single operation. The baseline data and tumor characteristics of patients are shown in Table 1.

**Table 1:** Comparison of baseline data between the two groups

Variable	SIB-IMRT group (n=34)	SEQ-IMRT group (n=32)	χ^2^	P
Age, years			0.000	0.988
≤ 45	18	17		
> 45	16	15		
Tumor size			0.000	0.988
< 20 mm	16	15		
≥ 20 mm	18	17		
Breast side			0.051	0.822
Left	19	17		
Right	15	15		
T-staging			0.229	*0.632*
T_1_	15	16		
T_2_	19	16		
N staging			0.490	*0.484*
N_0_	24	25		
N_1_	10	7		
Tumor characteristics			0.158	*0.691*
Invasive ductal carcinoma	28	27		
Invasive lobular carcinoma	3	2		
Ductal carcinoma	2	2		
Other	1	1		

### Comparison of the incidence of radiotherapy - related adverse reactions

Under the two radiotherapy methods, there were 25 patients with acute skin injury in both SIB-IMRT group and SEQ-IMRT group, accounting for 73.53% and 78.12% of the two groups, respectively. From the perspective of advanced skin injury, there were 2 patients with grade I reaction in both SIB-IMRT group and SEQ-IMRT group, accounting for 5.88% and 6.25% of the two groups respectively. Though the incidence of radioactive skin injury in the SEQ-IMRT group was slightly higher than the SIB-IMRT group, there was no statistical difference in the two groups (P > 0.05).

From the data of myelosuppression, 14.71% patients in the SIB-IMRT group had no myelosuppression, which was higher than the SEQ-IMRT group (12.5%). The incidence of grade I myelosuppression was 52.94%, slightly lower than the SEQ-IMRT group (50%). Nevertheless, the incidence rate of grade II and III myelosuppression in the SEQ-IMRT group was higher than the SIB-IMRT group. Overall, no significant difference were found between the two groups (P > 0.05).

Two patients in the SIB-IMRT group and the SEQ-IMRT group had Grade I radiation lung injury, accounting for 5.88% and 6.25% respectively, the former was slightly lower (P > 0.05). No radiation-induced lung injury greater than or equal to grade II occurred in both groups.

### Comparison of cosmetic effects in patients

In terms of cosmetic effects, 20 patients in the SIB-IMRT group were evaluated as “excellent” in the cosmetic effect of breast skin, and the fine rate of this group was 88.24%. In the SEQ-IMRT group, the cosmetic effect of breast skin was evaluated as “excellent” in 18 patients, and the fine rate of breast cosmetic effect in this group was 78.13%. There were 2 cases of poor cosmetic effect in both groups, accounting for 5.88% and 6.25% in the SIB-IMRT group and the SEQ-IMRT group, respectively. These results indicated that the cosmetic effect of patients in the SIB-IMRT group was better than the SEQ-IMRT group, but it did not reach the statistically significant level (P > 0.05) (Table 3).

**Table 3:** Comparison of cosmetic effects between the two groups

Group	Excellent	Good	General	Poor	Fine rate
SIB-IMRT group (n=34)	20 (58.82)	10 (29.42)	2 (5.88)	2 (5.88)	30 (88.24)
SEQ-IMRT group (n=32)	18 (56.25)	7 (21.87)	5 (15.63)	2 (6.25)	25 (78.13)
*χ* ^2^					3.580
*P*					*0.580*

### Comparison of long-term follow-up

There was no local recurrence in the SIB-IMRT group, the progression-free survival rate and the overall survival rate were all 100%. In the SEQ-IMRT group, local recurrence rate was 3.13%, the progression-free survival and overall survival rates were rate was 96.88% and 100%, respectively. No significant difference in 5-year progression-free survival rate and overall survival rate were found in the two groups (P > 0.05).

## Discussion

Breast cancer is the most common cancer and the primary reason for cancer deaths among women worldwide 19. With the continuous development of medical technology and the popularization of routine physical examination, more and more female patients are detected as early breast cancer patients in physical examination and treated in hospital. Women diagnosed with early- breast cancer and receiving lumpectomy generally receive WBRT after operation, which can reduce the recurrence and metastasis rate, hence making lumpectomy has the same effect as mastectomy 20. Due to the ongoing development of surgery and radiotherapy in BCS and the increasing conduct of plastic surgery, it is important to continuously evaluate the cosmetic results 21. The most fundamental purpose of breast preservation is to maintain the patient's good body shape and achieve the therapeutic effect without affecting the appearance. Fisher et al. 22 found that breast cancer is one kind of systemic disease with irregular metastasis of cancer cells. Although regional lymph nodes play an important role in immunity, they are not effective barriers for cancer cell filtration. However, the tumor diameter of early breast cancer is less than 3 cm, and there is no metastasis or only small metastasis in ipsilateral axillary lymph nodes, and no distant metastasis. The above views and clinical characteristics are the theoretical basis and indications for BCS for early breast cancer. In developed countries such as Europe and America, BCS can ensure the survival rate of patients, besides, maintaining the physiological function and appearance of the postoperative body, so it becomes a routine protocol for early breast cancer treatment 23. Postoperative WBRT is an important part of breast conserving therapy for early breast cancer, including conventional tumor bed sequential infusion IMRT and synchronous infusion IMRT.

In this paper, the clinical data of 66 patients were analyzed to compare the difference between the two radiotherapy methods. Among them, SEQ-IMRT group adopted the treatment plan of tumor bed sequential addition of IMRT, which showed that this method was time-consuming, had many radiotherapy times, poor patient compliance and high treatment cost, and could not meet patients' requirements for cosmetic effects and high quality of life. The tumor bed simultaneous IMRT regimen in SIB-IMRT group could significantly shorten the treatment cycle, and the adverse reactions incidence was reduced compared with that in SEQ-IMRT group, but no significantly difference in the two groups. Donovan et al. 24 found that the SIB-IMRT regimen reduced late toxicity compared to the SEQ-IMRT regimen. Two other studies comparing the clinical effects of SEQ and SIB also found that SIB treatment reduced acute dermal toxicity 25,26. No statistically significant differences in local recurrence rate, progression-free survival, as well as overall survival rate between the two groups in the current study, which was similar to the results of a retrospective comparative analysis of SEQ and SIB involving 126 patients 27. Both the ELIOT trial reported by Veronesi 28, and the TARGIT-A trial reported by Vaidya 29 showed that intraoperative and postoperative radiotherapy had similar overall survival rates for early breast cancer, which was the same as the conclusion of this study. In addition, the cosmetic effect in SIB-IMRT group was better than the SEQ-IMRT group. SIB-IMRT is more convenient and more efficient than SEQ-IMRT. From the patient's perspective, the treatment in the SIB-IMRT group makes the treatment of early breast cancer patients more reasonable and individualized, with higher patient compliance and less cost. Then from a socioeconomic perspective, fewer treatment fractions also lead to time and cost savings as well as reducing the workload of heath care providers. Besides, from the radiobiological point of view, reduction in overall treatment time is assumed to reduce the risk of tumor recurrence during the late phase of radiation treatment and probably to improve tumor control probability.

## Conclusion

To sum up, compared with the tumor bed SEQ-IMRT, SIB-IMRT treatment can significantly shorten the total radiotherapy time, reduce the number of patients' trips to the hospital, reduce treatment costs, and improve patient compliance under the condition of ensuring good survival conditions. In addition, while ensuring curative and cosmetic effects, there is no increase in adverse reactions of acute and late radiotherapy, and patients can complete radiotherapy as planned, ensuring the local control rate of tumor, which has a good application prospect. Whereas, our study also has some disadvantages. The sample size and follow-up time of this study are limited, and the detailed information during the sample collection process was incomplete, such as the specific location of the primary lesion not being counted. The statistics of some adverse reactions in the results were not comprehensive enough, such as the odds of developing edema, hyperpigmentation, fat necrosis and pain were not included in the statistics. The above indicators, late toxicity, and long-term efficacy need further exploration.

## Figures and Tables

**Table 2:** The occurrence of adverse reactions to radiotherapy after BCS in two groups

Adverse reactions	SIB-IMRT group (n=34)	SEQ-IMRT group (n=32)	χ^2^/Z	*P*
Acute skin reaction			0.524	0.600
Grade 0	9 (26.47)	7 (21.88)		
Grade I	17 (50.00)	16 (50.00)		
Grade II	8 (23.53)	9 (28.12)		
Advanced skin reaction			0.004	*0.950*
Grade 0	32 (94.12)	30 (93.75)		
Grade I	2 (5.88)	2 (6.25)		
Myelosuppression			0.435	*0.664*
Grade 0	5 (14.71)	4 (12.50)		
Grade I	18 (52.94)	16 (50.00)		
Grade II	9 (26.47)	10 (31.25)		
Grade III	2 (5.88)	2 (6.25)		
Radiation-induced lung injury			0.004	*0.950*
Grade 0	32 (94.12)	30 (93.75)		
Grade I	2 (5.88)	2 (6.25)		

**Table 4:** The long-term follow-up result was compared in the two groups

Group	Local recurrence	Progression-free survival	Overall survival
SIB-IMRT group (n=34)	0 (0)	34 (100)	34 (100)
SEQ-IMRT group (n=32)	1 (3.13)	31 (96.88)	32 (100)
χ^2^	1.079	1.079	0.000
*P*	*0.299*	*0.299*	1.000

## Data Availability

The datasets used and/or analyzed during the current study are available from the corresponding author on reasonable request.
